# Microsurgical Bypass for Complex Intracranial Aneurysms in the Endovascular Era: Insights from a High-Volume Referral Center

**DOI:** 10.3390/jcm14176027

**Published:** 2025-08-26

**Authors:** Yasmin Sadigh, Eva Joëlle Haasdijk, Ruben Dammers, Victor Volovici

**Affiliations:** 1 Department of Neurosurgery, Erasmus MC Stroke Center, Erasmus MC University Medical Center, 3015 GD Rotterdam, The Netherlands; 2 Center for Complex Microvascular Surgery, Erasmus MC University Medical Centre, 3015 GD Rotterdam, The Netherlands

**Keywords:** subarachnoid hemorrhage, unruptured aneurysms, aneurysm recanalization, clip reconstruction, aneurysm trapping

## Abstract

**Background/Objectives**: Endovascular treatment has become the primary treatment for intracranial aneurysms, yet direct bypass surgery remains an option in selected cases where standard approaches fail. This study aims to evaluate the role, indications, and outcomes of bypass surgery for intracranial aneurysm management in the current endovascular era. **Methods**: A single-center retrospective analysis was conducted on consecutive cases who underwent direct intracranial bypass surgery for intracranial aneurysms between 2015 and 2024. Data on demographics, aneurysm characteristics, indications, bypass type, patency, and clinical outcomes (using the modified Rankin Scale) were collected. **Results**: Of the 101 bypasses performed between 2015 and 2025, 25 were used for complex aneurysm cases. Intracranial bypass was necessary in as many as 5% of all microsurgical aneurysm repairs in 2023 and 10% in 2024. Bypass surgery was indicated in young patients with complex aneurysms not amenable to endovascular therapy (45%) and in 20% of the cases for recanalized aneurysms after previous endovascular repair. Intraoperative and postoperative bypass patency was confirmed for all patients except one case due to ongoing malignant brain swelling after an ongoing infarction. At follow-up, 87% of patients, with both ruptured and unruptured aneurysms, had a good outcome (mRS ≤ 2), and all patients had a patent bypass. Permanent morbidity was observed in 5% and procedure-related mortality in 0%. **Conclusions**: While bypass surgery constitutes a minority of intracranial aneurysm treatment by volume, its role in intracranial aneurysm repair is crucial and relevant in response to the evolving complexity of aneurysms. Further refinement of techniques is necessary.

## 1. Introduction

Intracranial aneurysms affect approximately 3–5% of the adult population, with a risk of rupture leading to subarachnoid hemorrhage (SAH) [1]. SAH is a type of hemorrhagic stroke associated with high mortality rates and severe neurological deficits in survivors [1]. Regarding the high morbidity and mortality associated with ruptured aneurysms, safe, effective, and durable treatment strategies are critical in improving patient outcomes [2]. For decades, microsurgical clipping was the mainstay of treatment for intracranial aneurysms. However, the development of minimal invasive endovascular techniques—such as coil embolization and flow diversion—has shifted the paradigm in strategies of both ruptured and unruptured aneurysm management [3,4].

Despite the success of endovascular techniques for aneurysmal control, these are not suitable to treat every case. Particularly in the treatment of complex aneurysms (not amendable to straightforward endovascular options or microsurgical clip reconstruction), bypass surgery may be an essential go-to technique for aneurysm exclusion and flow replacement [5,6,7,8].

Although bypass surgery continues to play a recognized role in the treatment of complex intracranial aneurysms, the precise indications for its use remain a subject of clinical debate [9,10,11]. Notably, the lack of benefit observed in major randomized trials—focused solely on bypass for steno-occlusive disease—has been inappropriately extrapolated to other indications, including aneurysm management [9,10,11]. This has, in some cases, led to the premature dismissal of bypass surgery as a therapeutic option in selected aneurysm cases where it may remain essential.

This study aims to provide a comprehensive overview of the cases in which bypass surgery was chosen as a treatment modality for intracranial aneurysm management in a high-volume center in Europe, where the bypass program commenced ten years ago.

## 2. Materials and Methods

### 2.1. Eligibility Criteria

Consecutive patients in our institution who underwent direct intracranial bypass surgery for aneurysmal management between 2015 and 2025 were retrospectively reviewed. This study was performed in accordance with the Preferred Reporting Of CasESeries in Surgery (PROCESS) guidelines [12].

The decision regarding treatment strategy is always taken in a biweekly multidisciplinary setting, in which a vascular neurosurgeon, neurointerventional radiologist, and stroke neurologist are present. Two vascular neurosurgeons perform all bypass surgeries, most often together (VV and RD). Any aneurysm surgery implies preparing the STA or OA graft for a potential bypass, and preparations beforehand are made to have potential bypass options available in any microvascular reconstruction case.

### 2.2. Data Collection

Data were collected and screened by two researchers (Y.S., E.J.H.) from electronic patient records. Disagreements were resolved through discussion with the senior author (V.V.). Data were independently checked by the senior author. Patient data such as age at treatment, sex, rupture status, World Federation of Neurosurgical Societies (WFNS) grading scale at presentation [13], The Modified Fisher grade (mFISHER) [14] for ruptured aneurysms, aneurysm location, size, morphology, previous treatment of aneurysm (if any), indication for bypass surgery, type of bypass (Donor–Receiver), and method of aneurysm exclusion were extracted.

Bypass patency was measured intraoperatively via administration of indocyanine green (ICG). Bypass patency was monitored using Computed Tomography Angiography (CTA) at 24 h, 7 days, and 21 days postoperatively. During follow-up, either CTA, Magnetic Resonance Angiography (MRA), or Digital Subtraction Angiography (DSA) was used for evaluating bypass patency. According to our postoperative protocols, strict blood pressure targets were set at 120 (systolic pressure) for low-flow bypasses for 72 h and for high flow for five days. Patients received antiplatelet therapy (Acetylsalicylic acid) for three months postoperatively to maintain bypass patency.

Postoperative neurological status was retrospectively reconstructed by one researcher (E.J.H.) and independently evaluated by a second researcher, using the modified Rankin Scale (mRS) [15]. Follow-up mRS were retrospectively constructed based on the performed neurological examination at discharge, at six months, and last available clinical follow-up. Data on procedure-related hypoperfusion (signs of procedure-related decreased perfusion and/or ischemia, established on Computed Tomography [CT] or perfusion imaging), permanent morbidity (permanent postoperative neurological deficits, unresolved after six months of follow-up), and mortality were also extracted.

### 2.3. Statistical Analysis

Descriptive statistics were used to report baseline demographics and clinical and patency outcomes. Statistical analyses were performed using SPSS software (IBM SPSS Statistics for Windows, Version 29.0.1.0.) and Rstudio, version 4.2.3.

## 3. Results

### 3.1. Patient Inclusion and Baseline Demographics

A total of 101 bypasses were performed at our institute in a 10-year period. Twenty-five bypasses were used in complex aneurysm cases (Table 1). In 2023, the total case volume for endovascular treatment was 83, for microsurgical repair was 59, and for bypass surgery was 3. In 2024, the total case volume for endovascular treatment was 98, microsurgical repair was 55, and bypass surgery was 6. While the yearly case volume was low in the initial years after the start of the program, it rose to 5% of all aneurysm microsurgical repairs in 2023 and 10% in 2024.

The median age at treatment was 59 years (Interquartile Range [IQR] 45.5–64.5), and the majority were female (*n* = 14; 70%). In total, ten patients (50%) presented with ruptured aneurysms (seven due to dissection, one due to a rupture during endovascular treatment). Of the unruptured aneurysms that were treated with bypass surgery, four harbored a recanalized aneurysm after previous endovascular treatment (Table 1).

**Figure 1:**
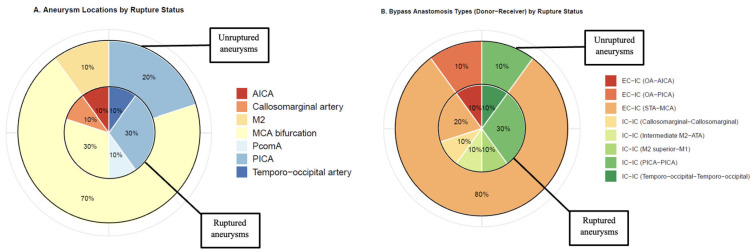
(**A**) Distribution of aneurysm locations per aneurysm rupture status; inner chart: ruptured aneurysms (*n* = 10); outer chart: unruptured or recanalized aneurysms (*n* = 10). (**B**) Distribution of bypass type (Donor–Receiver) per aneurysm rupture status; inner chart: ruptured aneurysms (*n* = 10); outer chart: unruptured or recanalized aneurysms (*n* = 10). M2: Second segment of the Middle Cerebral Artery, ATA: Anterior Temporal Artery, PcomA: Posterior Communicating Artery, PICA: Posterior Inferior Cerebellar Artery, IC-IC: Intracranial–Intracranial, EC-IC: Extracranial–Intracranial, STA: Superficial Temporal Artery, OA: Occipital Artery, AICA: Anterior Inferior Cerebellar Artery.

Of the patients with ruptured aneurysms, three (30%) cases harbored Middle Cerebral Artery (MCA) bifurcation aneurysms and three (30%) Posterior Inferior Cerebellar Artery (PICA) aneurysms (Table 1, Figure 1A). In unruptured or recanalized aneurysms, seven cases (70%) had MCA bifurcation aneurysms and two (20%) had PICA aneurysms. In total, four (20%) cases had giant (>25 mm), four (20%) cases had large (11–25 mm), and eight (40%) had small or blood-blister-like aneurysms (Table 1). Eight (40%) aneurysms were partially thrombosed, and six (30%) had a calcified base.

### 3.2. Indication for Bypass Surgery and Treatment Strategy

In nine (45%) cases, the aneurysm configuration appeared not amenable to endovascular therapy (Table 1). In three (15%) cases, bypass was indicated due to the need for parent vessel occlusion. Three cases (15%) were indicated for bypass surgery for MCA distal branch rescue.

Six (30%) aneurysms were trapped, and two (10%) were trapped and coiled after parent vessel occlusion (Table 1). Clip reconstruction was used in eight (40%) cases for aneurysm exclusion. In four (20%) patients, excision-reanastomosis of the parent vessel was performed. In twelve (60%) cases, Extracranial–Intracranial (EC-IC) bypass was used (ten cases with Superficial Temporal Artery [STA]-MCA anastomoses and two cases with Occipital Artery [OA]-Posterior Inferior Cerebellar Artery [AICA] anastomoses). Four cases (20%) received double-barrel STA-MCA anastomoses, and one (5%) case received double-barrel OA-AICA anastomoses. In cases with unruptured or recanalized aneurysms, the majority (*n* = 9, 90%) received an EC-IC bypass (8 STA-MCA, 1 OA-AICA) (Table 1, Figure 1B).

### 3.3. Aneurysm Occlusion, Bypass Patency, and Clinical Outcome

In all cases, complete aneurysm occlusion was achieved (Table 2). In nineteen (95%) cases, the anastomoses were patent intraoperatively, and in case 12, the bypass was occluded during intraoperative ICG videography. All anastomoses except case 12 remained patent postoperatively. Case 12 was taken to the OR after an intra-procedural aneurysm rupture during aneurysm re-coiling. A hemicraniectomy and attempt at microsurgical revascularization were undertaken. Due to progressive swelling, the bypass was not possible.

**Figure 2:**
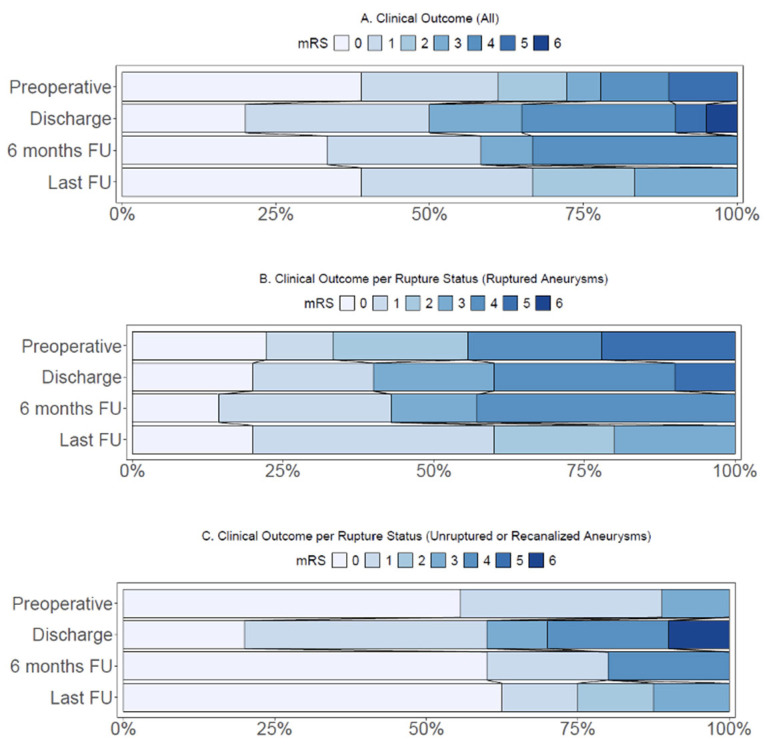
(**A**) Distribution of modified Rankin Score (mRS) in all cases (*n* = 20). (**B**) Distribution of modified Rankin Score in cases with ruptured aneurysms (*n* = 10). (**C**) Distribution of modified Rankin Score in cases with unruptured or recanalized aneurysms (*n* = 10). FU: Follow-up.

At last follow-up, with a median follow-up duration of 13 months (IQR 10–33), three (17%) cases had an mRS score of three or higher. In cases presenting with ruptured aneurysms, two (20%) cases presented with a poor mRS score at last follow-up (Table 3, Figure 2B). One case with an unruptured giant aneurysm had a poor mRS score (12%) at last follow-up (Table 3, Figure 2C).

### 3.4. Illustrative Cases

#### 3.4.1. Case 14—Giant Thrombosed MCA Bifurcation Aneurysm

**Figure 3:**
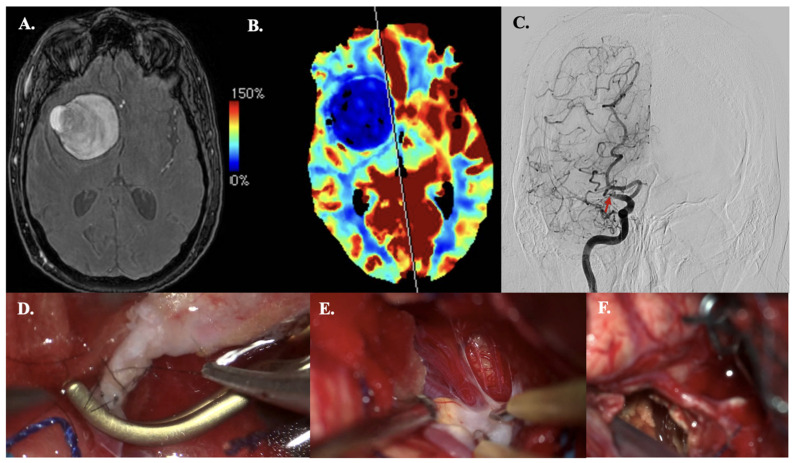
Illustrative Case #14. 63-year-old male with giant (>50 mm), thrombosed MCA bifurcation aneurysm with M1 occlusion. (**A**) Time of Flight MRA sequence of giant thrombosed aneurysm. (**B**) CT perfusion (Cerebral blood flow) demonstrating right hemisphere MRA territory perfusion deficit. (**C**) DSA demonstrating M1 occlusion (red arrow) and collateral formation, no aneurysm sac filling. (**D**) STA-M3 anastomosis. (**E**) The M1 and aneurysmal neck with severe atheromatous plaque and calcifications. (**F**) A clip is used to trap the M1 segment, and embolectomy is performed inside the aneurysmal lumen with the CUSA. The wall of the aneurysm remains in situ.

A 63-year-old man with a history of hypertension and atrial fibrillation presented with sudden loss of consciousness, left-side hemiparesis, and a spastic gait. MRA revealed a giant thrombosed MCA bifurcation aneurysm (size > 50 mm) accompanied by M1 occlusion and collateral formation (Figure 3A). CT perfusion revealed a right MCA territory perfusion deficit (with normal cerebral blood volume levels) (Figure 3B). DSA demonstrated no filling of the aneurysm from M1 but did demonstrate collateral filling from the Anterior Cerebral Artery and Posterior Cerebral Artery (Figure 3C). Microsurgical decompression of the aneurysm and revascularization to the hypoperfused areas were indicated due to the symptoms of mass effect and hemodynamic Transient Ischemic Attacks (TIAs). The latter was based on MCA territory hypoperfusion and insufficient collaterals from the posterior circulation, confirmed on preoperative CT perfusion (Figure 3B). Intraoperatively, the STA had a cut-flow of 60 mL·min^−1^. Before the anastomosis was performed, ICG videography showed a delayed time to drain of 11 s. After a STA-M3 bypass (Figure 3D–F) was performed, ICG videography confirmed bypass patency. The time to drain decreased to 7 s, and the STA had a measured flow of 30 mL·min^−1^. The aneurysm was then trapped proximally and distally. The thrombosed aneurysm sac was debulked to reduce mass effect. Postoperative CTA confirmed bypass patency. During hospitalization, he demonstrated transient mild hemiparesis and mild oculomotor palsy. Both were resolved within two months postoperatively.

#### 3.4.2. Case 15—PICA Dissection Fusiform Aneurysm

**Figure 4:**
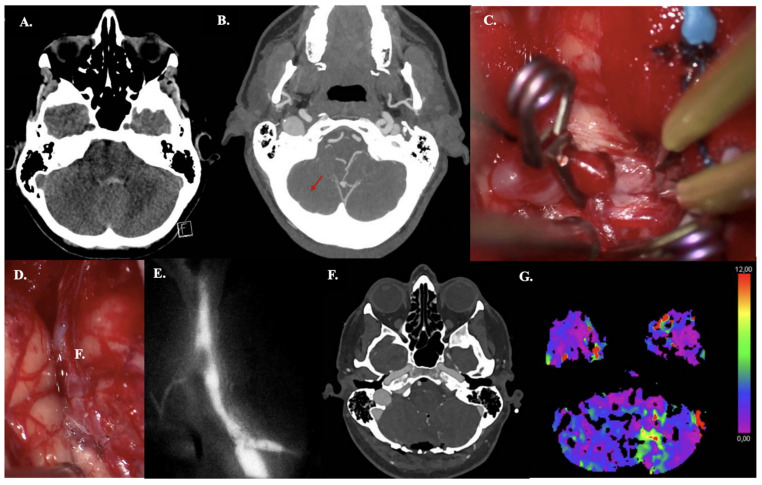
Illustrative Case #15. 44-year-old female with aSAH caused by blood blister (4 mm) dissected PICA aneurysm with intraventricular hemorrhage. (**A**) CT scan demonstrating mFISHER grade II. (**B**) Maximum intensity projection of ultra-high-definition CTA, demonstrating PICA aneurysm (red arrow). (**C**) Postoperative CTA confirming bypass patency. (**D**) Pilot clip on the dissecting aneurysm on the right tonsil and one clip on the outflow. No clip reconstruction possibilities available. (**E**) Aneurysm was excised, and the anastomosis was performed. (**F**) ICG video angiography confirms patency of the bypass. (**G**) Postoperative left cerebellar perfusion deficit (increased Tmax, but no increased MTT, or lower CBF or CBV), due to postoperative contusion.

A 44-year-old woman presented with acute headache and neck pain (WFNS grade II). CT demonstrated aneurysmal subarachnoid hemorrhage (aSAH) (mFisher grade II, based on thin aSAH and intraventricular hemorrhage) (Figure 4A). This aSAH was caused by a dissected PICA aneurysm (fusiform, dissected, size: 4 mm), confirmed on CTA (Figure 4B). An IC-IC (end-to-end) PICA reanastomosis and aneurysm excision were performed, and bypass patency was confirmed intraoperatively using ICG videography (Figure 4C–E). Due to aSAH-based hydrocephalus, an external ventricular drain was placed intraoperatively. Postoperative bypass patency was confirmed by CTA (Figure 4F). CT perfusion revealed minimal cerebellar perfusion deficits (only on Tmax images), corresponding to postoperative contusion (Figure 4G). The postoperative and follow-up course was uneventful.

#### 3.4.3. Case 19—Dissected Fusiform AICA Aneurysm

**Figure 5:**
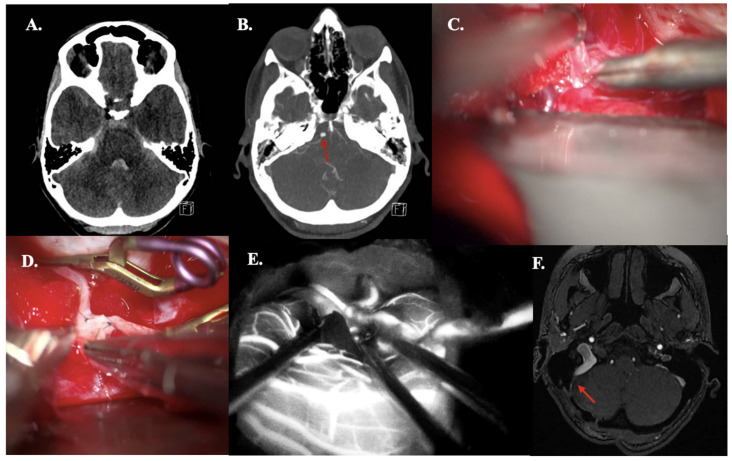
Illustrative Case #19. 35-year-old female with aSAH caused by fusiform (4 mm) dissected AICA aneurysm with intraventricular hemorrhage. (**A**) CT scan demonstrating mFISHER grade II. (**B**) Maximum intensity projection of ultra-high-definition CTA, demonstrating AICA aneurysm (red arrow). (**C**) The AICA was dissected just above the internal acoustic meatus. (**D**) The occipital artery—AICA anastomosis back wall was performed. (**E**) ICG videoangiography confirms patency of the bypass. (**F**) Five-month follow-up Time of Flight MRA confirming bypass patency (red arrow).

A 35-year-old woman presented with progressive headache and nausea with vomiting (WFNS grade II). CT demonstrated aneurysmal subarachnoid hemorrhage (mFisher grade II, based on thin aSAH and intraventricular hemorrhage) (Figure 5A). aSAH was caused by a dissected AICA aneurysm (fusiform, size: 4 mm), confirmed on CTA (Figure 5B). An OA-AICA bypass with endovascular parent vessel occlusion was performed. Intraoperative patency was confirmed using ICG videography (Figure 5C–E). At 3-month follow-up, bypass patency was confirmed by MRA, corresponding with the need for bypass maturation (Figure 5F). During hospitalization, she demonstrated abducens palsy, which completely resolved at the last follow-up.

## 4. Discussion

### 4.1. Key Findings

A total of twenty consecutive patients (25 anastomoses) who underwent direct bypass surgery for intracranial aneurysm treatment were included in this retrospective analysis. The median age at treatment was 59 years (IQR 45.5–64.5), with 70% of patients being female, and 50% presenting with ruptured aneurysms. Bypass surgery was indicated in 45% of cases due to the respective aneurysms not being amenable to endovascular treatment, with EC-IC bypass used in 60% of cases and IC-IC bypass in the rest. Bypass patency was observed in all cases at long-term follow-up. Good outcomes were observed in 87% of cases in both ruptured and unruptured aneurysms, with permanent morbidity in 5% and procedure-related mortality in 0%.

### 4.2. Role of Direct Bypass Surgery in Aneurysmal Management

Despite the endovascular advancements in treatment of intracranial aneurysms, bypass surgery continues to hold a vital, albeit highly specialized, role in the management of complex aneurysms that are not amenable to either endovascular or microsurgical clipping techniques alone [16,17]. More specifically, it serves as a flow replacement technique that allows safe trapping and parent vessel sacrifice of aneurysms. Additionally, it functions as a strategic adjunct, extending the armamentarium of the neurosurgeon to offer tailored, case-specific care where endovascular and standard microsurgical methods are insufficient.

Our institutional data aligns with outcomes in other high-volume vascular centers, highlighting that bypass surgery represents a small but essential fraction of all intracranial aneurysm treatments [18]. Its significance is further highlighted by an increasing case volume in recent years in our center. In 2023, bypass surgery accounted for 5% of microsurgically treated aneurysms, increasing to 11% in 2024, reflecting a growing recognition of its utility and expanding expertise to cases otherwise deemed untreatable. This higher volume of bypass surgery also aligns with the increase in complexity of cases referred for microsurgical treatment, as ‘standard’ cases are being successfully treated using endovascular techniques. However, when endovascular treatment is unsuccessful (e.g., due to aneurysm neck remnants or rebleed [19]), these cases may require a reconstructive approach that ensures definitive aneurysmal exclusion, while maintaining cerebral perfusion [17]. The occasional skepticism towards bypass surgery in aneurysm management stems partly from misconceptions based on earlier clinical trials focusing on bypass for steno-occlusive disease [9,10,11]. However, aneurysmal pathology and the goals of surgical revascularization in this setting are distinct, requiring a nuanced approach. Our findings and previous literature [5,6,7,8,16,17,18,20,21,22,23,24,25,26] show that bypass surgery remains an indispensable option in carefully selected cases, enabling durable aneurysmal exclusion and cerebral flow replacement [27].

### 4.3. Microvascular Training and Proficiency

The increasing role of direct bypass surgery in the management of complex intracranial aneurysms directly underscores the critical importance of dedicated microvascular training and surgical proficiency. Bypass surgery demands an advanced and maintained skill set with extensive hands-on experience to consistently achieve optimal outcomes [18]. Comprehensive training programs and multidisciplinary and international collaborative initiatives that emphasize microsurgical skills are essential to prepare neurosurgeons to meet these requirements [28,29]. Investment in microvascular training yields direct benefits not only in surgical outcomes but also in expanding the range of patients who can be offered bypass surgery as a viable treatment option. As such, the future of surgical aneurysm management is inherently linked to continued development and refinement of microsurgical education. Future microsurgical training programs should cultivate proficiency that matches the evolving complexity of aneurysmal pathology eligible for surgery. This approach ensures that cerebral revascularization techniques remain a safe and effective cornerstone of modern neurosurgical practice.

### 4.4. Starting from Scratch

One of the often-encountered questions in the minds of neurosurgeons practicing vascular neurosurgery in centers where intracranial bypass is not available is how to set up a bypass practice.

The first steps involve attending high-profile microvascular courses, such as those offered by the European Association of Neurosurgical Societies (EANS). These courses offer trainees, fellows, and neurosurgeons the opportunity to understand the entire spectrum of bypass possibilities as well as the current state-of-the-art indications. After attending courses, one should travel to centers where a bypass practice is flourishing in order to understand the flow and pre- and perioperative care for such patients. Before commencing the Rotterdam bypass program, the senior authors traveled to Japan and South Korea and maintained strong scientific ties with other high-volume centers.

Last but not least, research into further improving microvascular techniques and refining bypass practice, as well as its indication is paramount [30]. Centers such as the Erasmus MC in Rotterdam are both high volume in microvascular surgery as well as reference centers for microsurgical training.

### 4.5. Strengths and Limitations

The study focuses on cases of direct bypass surgery for intracranial aneurysms, offering valuable data for a small but critically important area of neurosurgery that is underrepresented in the current literature. It also reflects real-time neurosurgical decision-making regarding the treatment of complex aneurysms. This highlights the role of bypass surgery when endovascular and microsurgical options are insufficient or fail.

This study shares the inherent limitations associated with all retrospective case series. The mRS assessment was performed retrospectively, meaning that the given score was constructed based on a detailed and routine neurological assessment performed by clinicians. Also, the study reflects a highly selected cohort, which makes the findings less applicable for a broader patient population harboring intracranial aneurysms.

## 5. Conclusions

While direct bypass surgery constitutes a small portion of intracranial aneurysm treatments by volume, its role in this endovascular era is crucial and more relevant than ever in response to the evolving complexity of aneurysms referred to for microsurgical management. While patency and clinical outcomes are good for these complex lesions, continued refinement of patient selection criteria and surgical techniques is necessary to optimize its role in contemporary aneurysm treatment.

## Figures and Tables

**Table 1:** Patient and aneurysm characteristics and treatment (20 cases and 25 bypasses).

No.	Age at Treatment	Sex	Type bypass (Donor-Receiver)	Double Barrel	aSAH WFNS Grade	mFISHER Grade	Rupture Status	Location	Size and Morphology	Previous Treatments	Indication Bypass Treatment	Aneurysm Exclusion
1	58	M	EC-IC (STA-MCA)	Yes	-	-	Recanalized	MCA bif	Giant, saccular (partially thrombosed)	3x Coiled	Aneurysm rest	Clip reconstruction
2	67	F	IC-IC (Intermediate M2-ATA)	No	III	I	Ruptured	MCA bif	Small, saccular	None	Intermediate M2 branch from aneurysm sac	Clip reconstruction
3	32	F	IC-IC (Temporo-occipital-Temporo-occipital)	No	I	I	Ruptured, dissection	Temporo-occipital artery	Small, saccular	None	M4 branch rescue	Excision
4	69	F	EC-IC (STA-MCA)	Yes	-	-	Unruptured	MCA bif	Large, saccular (partially thrombosed, calcified base)	None	Not amenable to endovascular therapy	Clip reconstruction
5	61	F	EC-IC (STA-MCA)	No	II	III	Ruptured, dissection	MCA bif	Large, saccular	None	Not amenable to endovascular therapy	Clip reconstruction
6	65	F	EC-IC (STA-MCA)	Yes	-	-	Unruptured	MCA bif	Giant, saccular (partially thrombosed, calcified base)	None	Not amenable to endovascular therapy	Clip reconstruction
7	51	M	IC-IC (PICA-PICA)	No	I	II	Ruptured, dissection	PICA	Small, fusiform	None	Parent vessel (PICA) occlusion	Trapping, coiling
8	64	F	EC-IC (STA-MCA)	No	-	-	Unruptured	M2	Small, bilobar (partially thrombosed, calcified base)	None	Parent vessel (M2) occlusion	Clip reconstruction
9	48	F	EC-IC (OA-PICA)	No	-	-	Recanalized	PICA	Neck remnant, growth	1x Coiled	Neck remnant growth and recanalization	Trapping, coiling
10	45	F	IC-IC (M2 superior-M1)	No	V	I	Ruptured	MCA bif	Very large, saccular (partially thrombosed, calcified base)	None	M2 trunk occlusion	Excision
11	70	M	EC-IC (STA-MCA)	No	-	-	Unruptured	MCA bif	Large, saccular (partially thrombosed, calcified base)	None	Not amenable to endovascular therapy	Trapping
12	46	F	EC-IC (STA-MCA)	Yes	N/A	N/A	Ruptured	PcomA	Small, neck remnant	1x Coiled	Neck remnant rupture and ICA dissection during endovascular retreatment	Trapping
13	51	F	EC-IC (STA-MCA)	No	-	-	Recanalized	MCA bif	Large, saccular (partially thrombosed)	1x Flow Diverter	Aneurysm rest	Clip reconstruction
14	63	M	EC-IC (STA-MCA)	No	-	-	Unruptured	MCA bif	Giant, saccular (thrombosed, calcified base)	None	Not amenable to endovascular therapy	Trapping
15	44	F	IC-IC (PICA-PICA)	No	II	II	Ruptured, dissection	PICA	Blood-blister aneurysm	None	Not amenable to endovascular therapy	Excision
16	29	M	IC-IC (Callosomarginal-Callosomarginal)	No	V	III	Ruptured, dissection	Callosomarginal artery	Small, saccular	None	Traumatic aneurysm	Trapping
17	60	F	IC-IC (PICA-PICA)	No	I	II	Ruptured, dissection	PICA	Small, saccular	None	Not amenable to endovascular therapy	Excision
18	65	M	EC-IC (STA-MCA)	No	-	-	Unruptured	MCA bif	Giant, saccular (partially thrombosed)	None	Not amenable to endovascular therapy	Clip reconstruction
19	35	F	EC-IC (OA-AICA)	Yes	II	II	Ruptured, dissection	AICA	Very small, fusiform	None	Parent vessel (AICA) occlusion	Trapping
20	62	F	IC-IC (PICA-PICA)	No	-	-	Recanalized	PICA	Small neck remnant	2x coiled	Not amenable to endovascular therapy	Trapping

M: Male, F: Female, aSAH: Aneurysmal Subarachnoid Hemorrhage, WFNS: World Federation of Neurosurgical Societies, mFISHER: Modified Fisher grade, MCA bif: Middle Cerebral Artery Bifurcation, M2: Second segment of the Middle Cerebral Artery, ATA: Anterior Temporal Artery, PcomA: Posterior Communicating Artery, PICA: Posterior Inferior Cerebellar Artery, IC-IC: Intracranial–Intracranial, EC-IC: Extracranial–Intracranial, STA: Superficial Temporal Artery, OA: Occipital Artery, AICA: Anterior Inferior Cerebellar Artery. N/A: not available.

**Table 2:** Patency and clinical outcomes.

Patient	Aneurysm Occlusion	Intraoperative Bypass Patency	Postoperative Bypass Patency	FU Bypass Patency/FU Duration (mo)	mRS Discharge	mRS FU 6 Months	mRS Last Available FU/FU Duration (mo)	Procedure-Related Hypoperfusion	Procedure-Related Permanent Morbidity	Procedure-Related Mortality
1	Total occlusion	Yes	Yes	Yes/7	4	3	2/94	Yes	Yes	No
2	Total occlusion	Yes	Yes	Yes/1	3	N/A	1/2	No	No	No
3	Total occlusion	Yes	Yes	Yes/3	0	0	0/10	No	No	No
4	Total occlusion	Yes	Yes	Yes/4	1	N/A	1/13	No	No	No
5	Neck rest	Yes	Yes	Yes/5	1	1	0/24	No	No	No
6	Total occlusion	Yes	Yes	Yes/0	4	N/A	3/2	Yes	No	No
7	Total occlusion	Yes	Yes	Yes/21	3	3	2/58	No	No	No
8	Total occlusion	Yes	Yes	Yes/2	1	0	0/10	No	No	No
9	Total occlusion	Yes	Yes	Yes/6	0	0	0/54	No	No	No
10	Total occlusion	Yes	Yes	Yes/33	4	3	3/33	No	No	No
11	Total occlusion	Yes	Yes	Yes/0	3	N/A	N/A	No	No	No
12	Total occlusion	No	No	No	5	4	3/14	No	No	No
13	Total occlusion	Yes	Yes	Yes/15	0	1	0/12	No	No	No
14	Total occlusion	Yes	Yes	Yes/4	1	0	0/6	No	No	No
15	Total occlusion	Yes	Yes	Yes/7	0	1	1/6	No	No	No
16	Total occlusion	Yes	Yes	Yes/1	2	2	1/6	No	No	No
17	Total occlusion	Yes	Yes	Yes/6	4	N/A	2	Yes	No	No
18	Total occlusion	N/A	Yes	Yes/4	1	N/A	0	No	No	No
19	Total occlusion	Yes	Yes	Yes/5	1	N/A	1	No	No	No
20	Total occlusion	Yes	Yes	Yes/0	6	-	-	No	No	No

mRS: Modified Rankin Scale, FU: Follow-up. N/A: not available.

**Table 3:** Clinical outcome per rupture status.

Time Point	mRS Category	No. Patients (All, %)	No. Patients (Ruptured, %)	No. Patients (Unruptured or Recanalized, %)
		*n* = 18	*n* = 9	*n* = 9
Preoperative	0	7 (39)	2 (22)	5 (55)
	1	4 (22)	1 (11)	3 (33)
	2	2 (11)	2 (22)	0 (0)
	3	1 (5)	0 (0)	1 (11)
	4	2 (11)	2 (22)	0 (0)
	5	2 (11)	2 (22)	0 (0)
		*n* = 20	*n* = 10	*n* = 10
Discharge	0	4 (20)	2 (20)	2 (20)
	1	6 (30)	2 (20)	4 (40)
	2	0 (0)	0 (0)	0 (0)
	3	3 (15)	2 (20)	1 (10)
	4	5 (25)	3 (30)	2 (20)
	5	1 (5)	1 (10)	0 (0)
	6	1 (5)	0 (0)	1 (10)
		*n* = 12	*n* = 7	*n* = 5
6 months FU	0	4 (33)	1 (14)	3 (60)
	1	3 (25)	2 (28)	1 (20)
	2	0 (0)	0 (0)	0 (0)
	3	1 (8)	1 (14)	0 (0)
	4	4 (33)	3 (43)	1 (10)
	5	0 (0)	0 (0)	0 (0)
	6	0 (0)	0 (0)	0 (0)
		*n* = 18	*n* = 10	*n* = 8
Median last FU duration (mo, IQR)		13 (10–33)	12 (10–54)	19 (10–33)
Last FU	0	7 (39)	2 (20)	5 (62)
	1	5 (28)	4 (40)	1 (12)
	2	3 (17)	2 (20)	1 (12)
	3	3 (17)	2 (20)	1 (12)
	4	0 (0)	0 (0)	0 (0)
	5	0 (0)	0 (0)	0 (0)
	6	0 (0)	0 (0)	0 (0)

## Data Availability

Data is available upon reasonable written request to the corresponding author, including a research proposal and a data sharing agreement.

## Abbreviations

The following abbreviations are used in this manuscript:

aSAH	Aneurysmal Subarachnoid Hemorrhage
WFNS	World Federation of Neurosurgical Societies
mFISHER	Modified Fisher grade
ICG	Indocyanine Green
CTA	Computed Tomography Angiography
MRA	Magnetic Resonance Angiography
DSA	Digital Subtraction Angiography
CT	Computed Tomography
IQR	Interquartile Range
MCA	Middle Cerebral Artery
M2	Second segment of the Middle Cerebral Artery
M3	Third Segment of the Middle Cerebral Artery
ATA	Anterior Temporal Artery
PcomA	Posterior Communicating Artery
PICA	Posterior Inferior Cerebellar Artery
IC-IC	Intracranial-Intracranial
EC-IC	Extracranial-Intracranial
STA	Superficial Temporal Artery
OA	Occipital Artery
AICA	Anterior Inferior Cerebellar Artery
mRS	Modified Rankin Scale
FU	Follow-up

## References

[1] Etminan N., Chang H.S., Hackenberg K., de Rooij N.K., Vergouwen M.D.I., Rinkel G.J.E., Algra A. (2019). Worldwide incidence of aneurysmal subarachnoid hemorrhage according to region, time period, blood pressure, and smoking prevalence in the population: A systematic review and meta-analysis. JAMA Neurol..

[2] Volovici V., Verploegh I.S., Satoer D., Vrancken Peeters N.J.M.C., Sadigh Y., Vergouwen M.D.I., Schouten J.W., Bruggeman G., Pisica D., Yildirim G. (2023). Outcomes Associated with Intracranial Aneurysm Treatments Reported as Safe, Effective, or Durable: A Systematic Review and Meta-Analysis. JAMA Netw. Open.

[3] Roy D., Milot G., Raymond J. (2001). Endovascular treatment of unruptured aneurysms. Stroke.

[4] Molyneux A.J., Kerr R.S., Yu L.M., Clarke M., Sneade M., Yarnold J.A., Sandercock P. (2005). International Subarachnoid Aneurysm Trial (ISAT) Collaborative Group. International subarachnoid aneurysm trial (ISAT) of neurosurgical clipping versus endovascular coiling in 2143 patients with ruptured intracranial aneurysms: A randomised comparison of effects on survival, dependency, seizures, rebleeding, subgroups, and aneurysm occlusion. Lancet.

[5] Spetzler R.F., Roski R.A., Schuster H., Takaoka Y. (1980). The Role of EC-IC in the Treatment of Giant Intracranial Aneurysms. Neurol. Res..

[6] Jafar J.J., Russell S.M., Woo H.H. (2002). Treatment of Giant Intracranial Aneurysms with Saphenous Vein Extracranial-to-Intracranial Bypass Grafting: Indications, Operative Technique, and Results in 29 Patients. Neurosurgery.

[7] Kai Y., Hamada J., Morioka M., Yano S., Mizuno T., Kuroda J., Todaka T., Takeshima H., Kuratsu J. (2007). Treatment Strategy for Giant Aneurysms in the Cavernous Portion of the Internal Carotid Artery. Surg. Neurol..

[8] Zhu W., Tian Y.L., Zhou L.F., Song D.L., Xu B., Mao Y. (2011). Treatment Strategies for Complex Internal Carotid Artery (ICA) Aneurysms: Direct ICA Sacrifice or Combined with Extracranial-to-Intracranial Bypass. World Neurosurg..

[9] EC/IC Bypass Study Group (1985). Failure of extracranial-intracranial arterial bypass to reduce the risk of ischemic stroke. Results Int. Randomized Trial. N. Engl. J. Med..

[10] Powers W.J., Clarke W.R., Grubb R.L., Videen T.O., Adams H.P., Derdeyn C.P., COSS Investigators (2011). Extracranial-intracranial bypass surgery for stroke prevention in hemodynamic cerebral ischemia: The Carotid Occlusion Surgery Study randomized trial. JAMA.

[11] Ma Y., Wang T., Wang H., Amin-Hanjani S., Tong X., Wang J., Tong Z., Kuai D., Cai Y., Ren J. (2023). Extracranial-Intracranial Bypass and Risk of Stroke and Death in Patients With Symptomatic Artery Occlusion: The CMOSS Randomized Clinical Trial. JAMA.

[12] Agha R.A., Sohrabi C., Mathew G., Franchi T., Kerwan A., O’nEill N., Thoma A., Beamish A.J., Noureldin A., Rao A. (2020). The PROCESS 2020 Guideline: Updating Consensus Preferred Reporting of Case Series in Surgery (PROCESS) Guidelines. Int. J. Surg..

[13] Sano H., Inamasu J., Kato Y., Satoh A., Murayama Y., Murayama Y. (2016). Modified World Federation of Neurosurgical Societies subarachnoid hemorrhage grading system. Surg. Neurol. Int..

[14] Frontera J.A., Claassen J., Schmidt J.M., Wartenberg K.E., Temes R., Connolly E.S., Macdonald R.L., Mayer S.A. (2006). Prediction of symptomatic vasospasm after subarachnoid hemorrhage: The modified Fisher scale. Neurosurgery.

[15] Bonita R., Beaglehole R. (1988). Recovery of motor function after stroke. Stroke.

[16] Matano F., Tanikawa R., Kamiyama H., Ota N., Tsuboi T., Noda K., Miyata S., Matsukawa H., Murai Y., Morita A. (2016). Surgical Treatment of 127 Paraclinoid Aneurysms with Multifarious Strategy: Factors Related with Outcome. World Neurosurg..

[17] Matsukawa H., Miyata S., Tsuboi T., Noda K., Ota N., Takahashi O., Takeda R., Tokuda S., Kamiyama H., Tanikawa R. (2018). Rationale for Graft Selection in Patients with Complex Internal Carotid Artery Aneurysms Treated with Extracranial to Intracranial High-Flow Bypass and Therapeutic Internal Carotid Artery Occlusion. J. Neurosurg..

[18] Yoon S., Burkhardt J.K., Lawton M.T. (2019). Long-Term Patency in Cerebral Revascularization Surgery: An Analysis of a Consecutive Series of 430 Bypasses. J. Neurosurg..

[19] Molyneux A.J., Birks J., Clarke A., Sneade M., Kerr R.S. (2015). The Durability of Endovascular Coiling versus Neurosurgical Clipping of Ruptured Cerebral Aneurysms: 18 Year Follow-up of the UK Cohort of the International Subarachnoid Aneurysm Trial (ISAT). Lancet.

[20] Aboukais R., Verbraeken B., Leclerc X., Gautier C., Vermandel M., Bricout N., Lejeune J.P., Menovsky T. (2019). Protective STA-MCA Bypass to Prevent Brain Ischemia during High-Flow Bypass Surgery: Case Series of 10 Patients. Acta Neurochir..

[21] Endo H., Fujimura M., Shimizu H., Endo T., Omodaka S., Inoue T., Sato K., Niizuma K., Tominaga T. (2020). Optimal Timing of Extracranial-Intracranial Bypass with Microsurgical Trapping for Ruptured Blister Aneurysms of the Internal Carotid Artery. World Neurosurg..

[22] Lam J., Ravina K., Rennert R.C., Russin J.J. (2021). Cerebrovascular Bypass for Ruptured Aneurysms: A Case Series. J. Clin. Neurosci..

[23] Murai Y., Matano F., Shirokane K., Tateyama K., Koketsu K., Nakae R., Sekine T., Mizunari T., Morita A. (2021). Lesion Trapping with High-Flow Bypass for Ruptured Internal Carotid Artery Blood Blister-Like Aneurysm Has Little Impact on the Anterior Choroidal Artery Flow: Case Series and Literature Review. World Neurosurg..

[24] Peeters S.M., Colby G.P., Kim W.J., Bae W.I., Sparks H., Reitz K., Tateshima S., Jahan R., Szeder V., Nour M. (2023). Proximal Internal Carotid Artery Occlusion and Extracranial-Intracranial Bypass for Treatment of Fusiform and Giant Internal Carotid Artery Aneurysms. World Neurosurg..

[25] Wolfswinkel E.M., Ravina K., Rennert R.C., Landau M., Strickland B.A., Chun A., Wlodarczyk J.R., Abedi A., Carey J.N., Russin J.J. (2022). Cerebral Bypass Using the Descending Branch of the Lateral Circumflex Femoral Artery: A Case Series. Oper. Neurosurg..

[26] Chen Y., Chen P., Duan G., Li R., Li Z., Guo G. (2023). Extracranial-Intracranial Bypass Surgery for Intracranial Aneurysm of the Anterior Cerebral Circulation: A Systematic Review and Meta-Analysis. Front. Neurol..

[27] Esposito G., Amin-Hanjani S., Regli L. (2016). Role of and Indications for Bypass Surgery After Carotid Occlusion Surgery Study (COSS)?. Stroke.

[28] Volovici V., Cenzato M., Meling T.R., EXPERVASC (2024). The European Expertise Network for Open Microvascular Surgery. Lancet Neurol..

[29] Volovici V., Verploegh I.S.C., Vos J.C., Delwel E.J., Bijvoet H.W.C., van Putten E.H.P., Schouten J.W., Avezaat C.J.J., Dirven C.M.F., Dammers R. (2020). Can young vascular neurosurgeons become proficient in microsurgical clip reconstruction in the endovascular era? A Rotterdam cohort spanning 2 decades with propensity score matching for complexity. World Neurosurg..

[30] Dindelegan G.C., Dammers R., Oradan A.V., Vinasi R.C., Dindelegan M., Volovici V. (2021). The Double Stitch Everting Technique in the End-to-Side Microvascular Anastomosis: Validation of the Technique Using a Randomized N-of-1 Trial. J. Reconstr. Microsurg..

